# Serial Changes of Circulating Tumor Cells in Patients with Hepatocellular Carcinoma Treated with Atezolizumab Plus Bevacizumab

**DOI:** 10.3390/cancers16132410

**Published:** 2024-06-29

**Authors:** Yosuke Murata, Takuto Nosaka, Yu Akazawa, Tomoko Tanaka, Kazuto Takahashi, Tatsushi Naito, Hidetaka Matsuda, Masahiro Ohtani, Yasunari Nakamoto

**Affiliations:** Second Department of Internal Medicine, Faculty of Medical Sciences, University of Fukui, Fukui 910-1193, Japan; yosukem@u-fukui.ac.jp (Y.M.); nosat@u-fukui.ac.jp (T.N.); aka0124@u-fukui.ac.jp (Y.A.); kawakami@u-fukui.ac.jp (T.T.); tkazuto@u-fukui.ac.jp (K.T.); naitot@u-fukui.ac.jp (T.N.); hidem@u-fukui.ac.jp (H.M.); mohtani@u-fukui.ac.jp (M.O.)

**Keywords:** immune checkpoint inhibitor, liquid biopsy, RNA sequencing, TGF-β signaling, apoptosis signaling

## Abstract

**Simple Summary:**

Circulating tumor cells (CTCs) derived from peripheral blood have a notable advantage in that they are noninvasive and real-time biomarkers and have attracted attention in monitoring therapeutic efficacy. Combination immunotherapy has promising outcomes in patients with unresectable hepatocellular carcinoma (HCC). However, there is no reliable biomarker for predicting disease progression. In this study, CTCs were serially collected from HCC patients undergoing atezolizumab plus bevacizumab (Atezo+Bev). Changes in CTC numbers during Atezo+Bev treatment reflected the tumor volume, and patients with elevated transforming growth factor (TGF)-β signaling molecules had a poorer response, whereas those with elevated apoptosis signaling molecules had a favorable response. This highlights that changes in the expression of TGF-β molecules of CTCs could serve as novel biomarkers for the early prediction of therapeutic response in patients with unresectable HCC undergoing Atezo+Bev.

**Abstract:**

Immune checkpoint inhibitors have promising outcomes in patients with hepatocellular carcinoma (HCC); however, there is no reliable biomarker for predicting disease progression. Circulating tumor cells (CTCs) derived from peripheral blood have attracted attention in monitoring therapeutic efficacy. In this study, CTCs were serially collected from HCC patients undergoing atezolizumab plus bevacizumab (Atezo+Bev), and changes in molecular expression and CTC numbers were analyzed to identify effective biomarkers. Changes in CTC numbers during Atezo+Bev reflected the tumor volume. Targeted RNA sequencing with next-generation sequencing (NGS) revealed that patients with elevated transforming growth factor (TGF)-β signaling molecules had a poorer response, whereas those with elevated apoptosis signaling molecules had a favorable response. In addition, compared with changes in CTC counts, changes in TGF-β signaling molecule expression in CTCs accurately and promptly predicted treatment response. Overall, NGS analysis of CTC-derived RNA showed that changes in TGF-β signaling molecules predict treatment response earlier than changes in CTC counts. These findings suggest that changes in the expression of TGF-β molecules in CTCs could serve as novel biomarkers for the early prediction of therapeutic response in patients with unresectable HCC undergoing Atezo+Bev.

## 1. Introduction

Hepatocellular carcinoma (HCC) is the sixth most commonly diagnosed cancer and the third leading cause of cancer-related mortality in the world [1]. Immune checkpoint inhibitors (ICIs) are more frequently employed in the treatment of patients with advanced-stage HCC. Notably, the combination of atezolizumab, a programmed death ligand 1 (PD-L1) inhibitor, plus bevacizumab, an antivascular endothelial growth factor (VEGF) antibody, has shown promising outcomes as a first-line systemic therapy for unresectable HCC [2,3]. However, there remains a critical need to identify reliable biomarkers to monitor disease progression and elucidate the resistance mechanisms affecting combination therapies [4].

Among the various blood-based tumor-derived materials, circulating tumor cells (CTCs) are shed from primary and/or metastatic tumors into the bloodstream [5]. CTCs, being viable tumor cells and amenable to serial sampling, have garnered attention for their clinical potential in monitoring treatment response and predicting prognosis [6,7]. CTCs exhibit significant heterogeneity in patients, and their genomic, transcriptomic, and epigenetic profiles offer avenues for exploration into tumor biology [5,8]. We hypothesized that sequential profiling of CTCs during atezolizumab plus bevacizumab (Atezo+Bev) therapy in patients with HCC would reveal molecular mechanisms of treatment effectiveness or resistance.

In this study, we aimed to identify CTC biomarkers capable of predicting treatment response by analyzing changes in the number and RNA expression of CTCs during Atezo+Bev treatment in patients with HCC. The number of CTCs measured by flow cytometry was changed, reflecting the effect of Atezo+Bev at the time of response evaluation. Moreover, the changes in expression of the TGF-β signaling molecules in CTCs accurately predicted the therapeutic effect earlier than the changes in CTC counts. Changes in expression of TGF-β molecules in CTCs may be a novel biomarker for the early predictors of therapeutic response to Atezo+Bev treatment in patients with unresectable HCC.

## 2. Materials and Methods

### 2.1. Study Protocol and Participants

This prospective study was conducted in accordance with the Declaration of Helsinki and was approved by the Research Ethics Committee of the University of Fukui (20210168). Written informed consent was obtained from all participants. Peripheral blood samples were collected from 10 healthy donors and 44 patients with HCC at the University of Fukui Hospital between September 2020 and August 2023. In 22 patients with unresectable HCC treated with Atezo+Bev, peripheral blood samples were collected at baseline, during the initial response evaluation, and at various follow-up time points.

### 2.2. Etiology of Liver Diseases

The etiology of HCC was determined in patients who were positive for the anti-hepatitis C virus antibody (HCV Ab) and were categorized as HCV-positive, while those testing positive for the surface antigen of the hepatitis B virus (HBV) (HBsAg) were classified as HBV-positive. Patients testing negative for both anti-HCV antibody and HBsAg were defined as non-B-non-C (NBNC) tumors.

### 2.3. Treatment Protocol

Atezo+Bev was administered following the IMbrave 150 protocol schedule, with doses of 1200 mg of atezolizumab and 15 mg/kg of bevacizumab given every 3 weeks until either clinical benefits were lost, or the toxicity was intolerable [2]. Adjustments to the dosage or discontinuation of Atezo+Bev were made to manage adverse events (AEs). Treatment response was assessed by dynamic computed tomography or gadolinium ethoxybenzyl magnetic resonance imaging (Gd-EOB-MRI) at 8–12 weeks after the initial administration. Treatment was discontinued if intolerable adverse events occurred or if there was evidence of progressive disease (PD). The Response Evaluation Criteria for Solid Tumors (RECIST) criteria ver. 1.1 was used to assess radiological responses. AEs were evaluated and graded in accordance with the National Cancer Institute Common Terminology Criteria for Adverse Events v5.0 (https://ctep.cancer.gov/protocoldevelopment/electronic_applications/ctc.htm, (accessed on 1 September 2020)).

### 2.4. Evaluation of the Treatment Response

Overall survival (OS) was defined as the time from the first treatment cycle to death. Progression-free survival (PFS) was defined as the duration from the first treatment cycle to either death or the detection of radiological evidence of tumor progression.

### 2.5. Enrichment of CTCs and RNA Extraction

CTCs were enriched from peripheral blood samples (10 mL each) using the RosetteSep Human CD45 Depletion Cocktail (StemCell Technologies, Vancouver, BC, Canada) with reference to previous literature [9]. The sample was incubated with the Depletion Cocktail, and separation of CTCs was performed in SepMate 50 mL tubes (StemCell Technologies) containing 15 mL of Lymphoprep density gradient medium (StemCell Technologies). After centrifugation for 5 min at 1200 rpm, the upper phase was poured into a 50 mL tube with PBS with fetal bovine serum (Sigma–Aldrich, St. Louis, MO, USA). Following centrifugation at 300× *g* for 10 min, the sample was lysed with ammonium chloride solution (StemCell Technologies). The cell pellets were then rinsed twice by 50 mL of PBS with 2% FBS. For each patient, the enriched cells were divided equally into two halves, with one half designated for flow-cytometric analysis and the other for RNA extraction for next-generation sequencing (NGS) and quantitative reverse transcription polymerase chain reaction (qRT-PCR). Total RNA was extracted with the RNeasy Mini Kit (Qiagen, Hilden, Germany). Both RNA extraction and flow-cytometric analysis were performed within 8 h following collection.

### 2.6. Flow-Cytometric Analysis

After blood separation using RosetteSep, enriched cells were labeled with fluorescent dye conjugated antibodies, including APC/Cyanine7 mouse anti-human CD45 (2D1) (BioLegend) and PE mouse anti-human pan-cytokeratin (cytokeratin 4, 5, 6, 8, 10, 13, and 18) (C-11) (Cayman chemical Ann Arbor, MI, USA). The cell pellets were analyzed by multiparametric flow cytometry with a BD FACSAriaII (BD Biosciences San Jose, CA, USA). Specifically, CD45 negative and Pan-CK positive cells were defined as CTCs. Data analysis was performed with FlowJo version 10.9.0 (BD Biosciences). Corresponding isotype control antibodies (BD Biosciences) were used for compensation (the list of antibodies used are provided in Table 1) and 7-aminoactinomycin D (7-AAD; BD Biosciences, San Jose, CA, USA) for dead cell elimination. 7-AAD(-)CD45(−)PanCK(+) populations were considered as CTCs.

### 2.7. Next Generation Sequencing

The total RNA extracted from the enriched cells with RosetteSep underwent quantification and integrity evaluation on a Qubit^®^ 2.0 Fluorometer (ThermoFisher Scientific, Waltham, MA, USA) using the RNA HS Assay (ThermoFisher Scientific) and the Bioanalyzer 2100 (Agilent Technologies, Palo Alto, CA, USA) for next-generation sequencing. Library construction commenced with 1–3 ng of RNA input. Library construction and complementary DNA (cDNA) synthesis were performed following the manufacturer’s instructions. In particular, AmpliSeq cDNA Synthesis for Illumina (Illumina, San Diego, CA, USA) was utilized for cDNA synthesis. For RNA library preparation, the AmpliSeq for Illumina custom RNA panel (Illumina), AmpliSeq library PLUS for Illumina (Illumina), and AmpliSeq CD indexes Set A for Illumina (Illumina) were employed, with the custom panel comprising 373 genes associated with cancer progression. RNA sequencing was conducted using the MiSeq (Illumina) with 2 × 150 bp paired-end runs using the MiSeq Reagent Kit v3 (Illumina) following the manufacturer’s instructions. The resulting FASTQ files from sequencing were subjected to automatic analysis using the Local Run Manager RNA Amplicon Analysis Module (Illumina). Normalization of read count information and identification of differentially expressed genes (DEGs) between baseline and treatment effect assessment was performed using the edgeR package, with unsupervised hierarchical clustering analysis conducted on DEGs (*p* < 0.05). Raw data were uploaded to the NCBI Gene Expression Omnibus (GEO) database (GSE261186). Functional annotation of the genes was carried out by the gene set enrichment analysis (GSEA) method [10], supported by the Broad Institute website (https://www.gsea-msigdb.org/gsea/index.jsp, (accessed on 1 November 2021)) and performed via GSEA software (version 4.3.2; Broad Institute, Inc., Massachusetts Institute of Technology, Boston, MA, USA, and Regents of the University of California, Oakland, CA, USA), where adjusted *p*-values < 0.05 were considered to indicate significant enrichment.

### 2.8. RNA Extraction and Nested PCR

Reverse transcription of total RNA was performed with the High-Capacity cDNA Reverse Transcription Kit (Applied Biosystems, Foster City, CA, USA) to synthesize first-strand cDNA, followed by qPCR performed with TaqMan Gene Expression Assay (ThermoFisher Scientific, Waltham, MA, USA) and StepOnePlus (Applied Biosystems, Carlsbad, CA, USA). Preamplification of the cDNA was performed with TaqMan PreAmp Master Mix (Applied Biosystems). The primers and TaqMan probes of these genes were selected from predesigned assays of Applied Biosystems (Table 2). In detail, each target initially underwent RT-PCR reaction using TaqMan^TM^ PreAmp Master Mix, with samples incubated at 95 °C for 10 min before undergoing 14 cycles of a two-step program (denaturation at 95 °C for 15 s and annealing at 60 °C for 4 min). This first reaction was performed on TaKaRa PCR Thermal Cycler Dice Standard (TaKaRa bio, Kusatsu, Japan). Subsequently, the samples served as templates for the second semi-nested RT-PCR amplification using StepOnePlus (Applied Biosystems) with reference to a previous study [11]. The expressions of the target genes were analyzed using the ΔΔCt comparative threshold method, with hypoxanthine phosphoribosyltransferase 1 (HPRT1) utilized as the internal control.

### 2.9. Statistical Analyses

Statistical significance was assessed by the Wilcoxon matched-pair rank test, Mann–Whitney U test, or one-way analysis of variance, followed by the Tukey–Kramer post hoc test. Cumulative survival was evaluated by the Kaplan–Meier analysis, and differences were determined by the log-rank test. All statistical analyses were carried out via GraphPad Prism software (version 10; GraphPad Software Inc., San Diego, CA, USA), and statistical significance was set at *p* < 0.05.

## 3. Results

### 3.1. Changes in CTC Counts during Atezo+Bev Treatment Reflect the Treatment Response in Patients with Unresectable HCC

**Figure 1:**
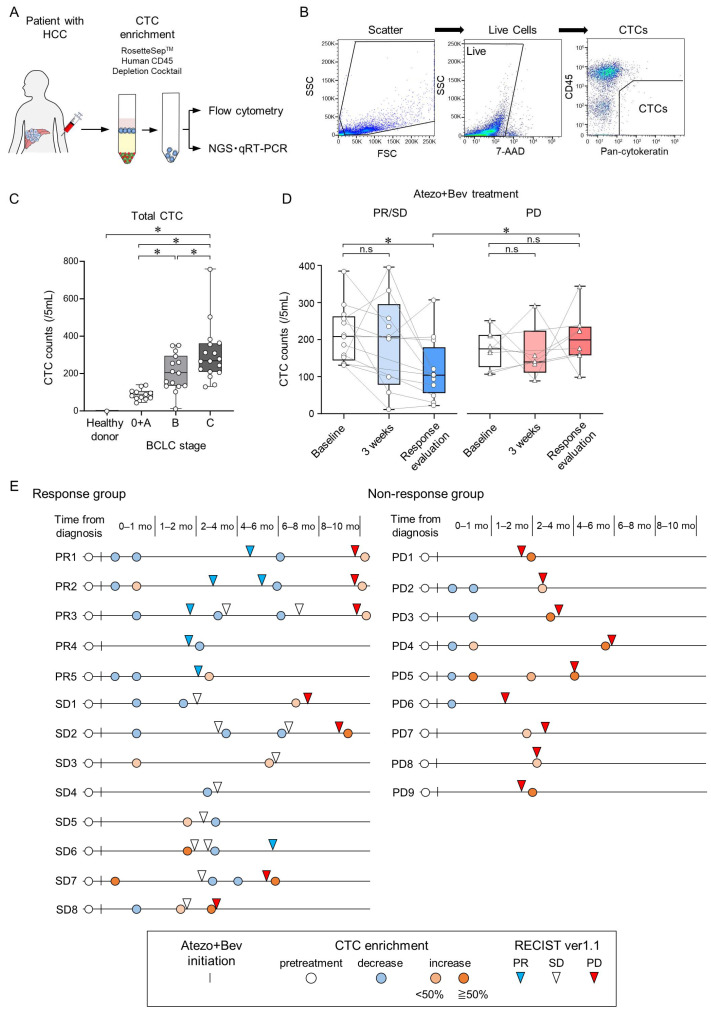
Changes in circulating tumor cell (CTC) counts during treatment with atezolizumab and bevacizumab in patients with unresectable HCC. (**A**) Peripheral blood samples were collected from patients, and CTCs were enriched using Rosettesep™. Subsequently, surface protein expression was analyzed by flow cytometry, while gene expression was analyzed using both NGS and qRT-PCR. (**B**) A flowchart illustrating the process of CTC isolation from patients with HCC via multiparametric flow cytometry. CTC analysis involved two steps: (i) removal of dead cells using 7-AAD and (ii) isolation of PanCK(+) CD45(−) cells, i.e., CTCs. (**C**) Total CTC counts in healthy controls (n = 6) and patients with HCC stratified by BCLC stage (BCLC stage 0/A/B/C; n = 6/6/16/16). (**D**) Changes in total CTC counts at baseline, 3 weeks after treatment initiation, and at the first response evaluation in patients with HCC undergoing Atezo+Bev therapy, categorized into PR/SD (n = 13) and PD (n = 9). (**E**) Swimmer plot depicting CTC counts in patients with HCC during Atezo+Bev treatment. Each patient is represented by an individual bar, with the left panel showing the response group (PR/SD) and the right panel showing the non-response group (PD). The date of the first treatment with Atezo+Bev is depicted by a vertical line. The chart shows the timing of CTC counts (pre-treatment; white circle; decrease; blue circle, <50% increase, pink circle; ≥50% increase, red circle). Treatment response evaluation using RECIST v1.1 (PR, inverse blue triangle; SD, inverse white triangle; PD, inverse red triangle) is also shown. (**C**,**D**) Box and whisker plots represent median values (center lines); box edges represent 25th and 75th percentiles; and the whiskers indicate the minimum and maximum values. (**C**) Tukey–Kramer post hoc test. (**D**) Wilcoxon matched-pairs signed rank test was used for within-group comparison. Mann–Whitney test was employed for between-group comparisons. * *p* < 0.05, n.s., not significant.

To accurately isolate CTCs from the peripheral blood of patients with HCC based on cell surface proteins, we constructed a CTC assay system involving flow cytometry analysis after CTC enrichment. For CTC enrichment, RosetteSep was used to deplete leukocytes and erythrocytes using a tetrameric antibody complex recognizing CD45, CD66b, and glycophorin A, followed by density gradient centrifugation (Figure 1A). The CD45(−)PanCK(+) population detected by flow cytometry was identified as CTCs (Figure 1B). In a cohort consisting of 6 healthy donors (HDs) and 44 patients with HCC, the number of CTCs in patients with HCC was significantly higher than in HDs (clinical background information is provided in Table 3 and Figure 1C). Furthermore, CTC counts in patients with HCC significantly correlated with the Barcelona Clinic Liver Cancer (BCLC) stages. Importantly, during the clinical course of Atezo+Bev treatment, CTC numbers at 3 weeks, an early time point post-treatment initiation, remained unchanged from baseline in both the PR/SD (patients who showed partial response or stable disease at first response evaluation) and PD groups (patients who showed progressive disease at first response evaluation). However, upon response evaluation, CTC numbers decreased from baseline in the PR/SD group, while no significant change was observed in the PD group (Figure 1D).

In the long-term course of Atezo+Bev treatment, within the response (PR/SD) group, CTC numbers decreased at the time of the initial response evaluation; however, they increased in patients with disease progression at subsequent response evaluations (Figure 1E). Conversely, in the non-response (PD) group, CTC numbers increased at the time of initial response evaluation. These findings suggest that the CTC numbers measured by this CTC assay system do not change at the 3-week time point, but accurately reflect changes in tumor burden at response evaluation in patients with unresectable HCC undergoing Atezo+Bev treatment.

### 3.2. Molecular Changes of CTCs Using NGS in Patients with HCC Treated with Atezo+Bev

**Figure 2:**
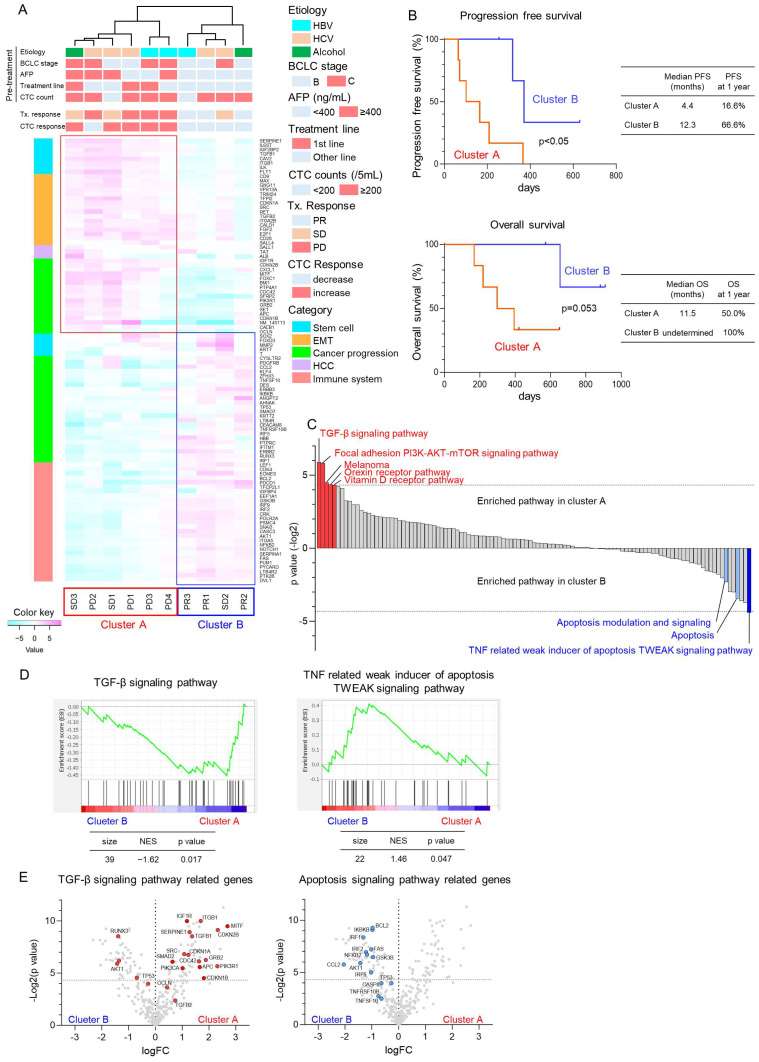
Molecular changes in CTCs using next-generation sequencing in patients with HCC treated with atezolizumab and bevacizumab. (**A**) Unsupervised hierarchical clustering analysis was conducted on 99 genes exhibiting significant changes in the expression of CTCs of patients with HCC (n = 10) before Atezo+Bev treatment and at the initial treatment response evaluation. Patients were categorized into two clusters: cluster A (n = 6) representing non-responders and cluster B (n = 4) representing responders to Atezo+Bev treatment. Pre-treatment patient characteristics, evaluation of initial treatment response, and changes in CTC counts are shown. The heatmap illustrates the relative expression variation levels of the 99 genes. (**B**) Kaplan–Meier curves illustrating progression-free survival and overall survival for patients in cluster A and B. (**C**) Results of the gene set enrichment analysis (GSEA) of the Wikipathways. The *p*-value (−Log2 ratio) for each gene set is shown. Red bars indicate gene sets enriched in cluster A with *p* < 0.05, while blue bars indicate gene sets enriched in cluster B with *p* < 0.05. Light blue bars denote apoptosis-related gene sets with *p* > 0.05. (**D**) Enrichment indexes of two gene sets depicted in the TGF-β signaling pathway (left panel) and TNF-related weak inducer of apoptosis (TWEAK) signaling pathway (right panel). Gene sets were considered significantly enriched at normalized enrichment score (NES) >1 or <−1 and nominal *p*-value < 0.05. (**E**) Volcano plots showing fold changes in expression and *p*-values of TGF-β signaling pathway-related genes (**left panel**) and apoptosis signaling-related genes (**right panel**) before Atezo+Bev treatment and at the initial response evaluation.

To evaluate the changes in molecular expression of CTCs in patients with HCC treated with Atezo+Bev, we designed an NGS panel comprising 373 genes associated with stem cell potency, epithelial–mesenchymal transition (EMT), HCC progression, and cancer immunotherapy (Table 4). Clinical background is provided in Table 5. Notably, the 99 genes exhibiting significant changes in expression in CTCs at the time of response evaluation compared to pre-treatment initiation were subjected to unsupervised hierarchical clustering analysis (Figure 2A). Patients with HCC were categorized into two clusters, with cluster A comprising four PD and two SD cases, whereas cluster B included three PR and one SD case. During treatment, CTC counts increased in five out of six patients in cluster A, whereas all four cases in cluster B demonstrated decreased CTC counts. The median PFS was significantly more prolonged in cluster B than in cluster A, with a tendency towards improved OS in cluster B (Figure 2B). GSEA revealed enrichment of the TGF-β signaling pathway in cluster A, and the apoptosis-related signaling pathway was enriched in cluster B (Figure 2C). Notably, genes associated with the TGF-β signaling pathway, including cyclin-dependent kinase inhibitor 2B (CDKN2B), growth factor receptor binding protein 2 (GRB2), and phosphoinositide-3-kinase regulatory subunit 1 (PIK3R1), were significantly enriched in cluster A (Figure 2D,E). Conversely, cluster B exhibited enrichment in the expression of genes associated with the apoptosis signaling pathway, such as the Fas cell surface death receptor (FAS) and interferon regulatory factor 1 (IRF1). These findings suggest that patients with elevated expression of genes related to TGF-β signaling in CTCs exhibit a poor therapeutic response, whereas those with elevated expression of genes related to the apoptosis signaling demonstrate favorable responses to Atezo+Bev treatment.

### 3.3. TGF-β Signaling-Related Gene Expression in CTCs Reflect Early Treatment Response to Atezo+Bev

**Figure 3:**
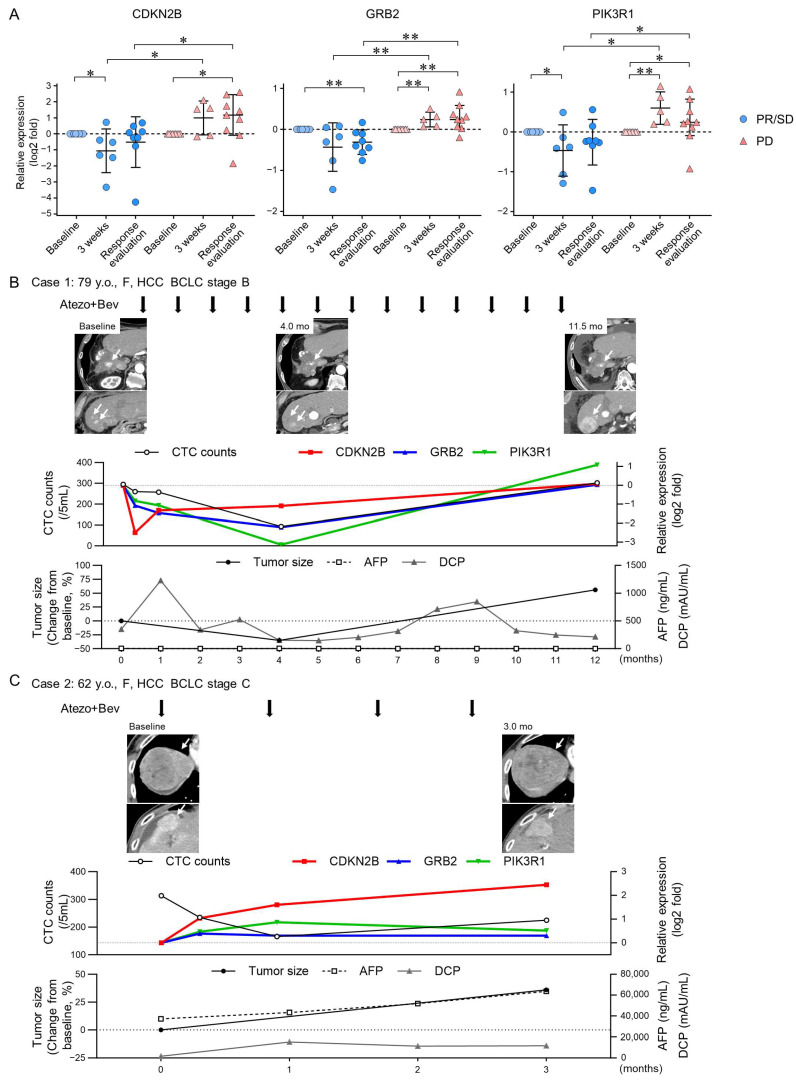
Changes in TGF-β signaling-related gene expression in CTCs of patients with HCC treated with atezolizumab and bevacizumab. (**A**) messenger RNA (mRNA) expression levels were analyzed via real-time quantitative reverse transcription polymerase chain reaction at baseline, 3 weeks post-treatment, and at response evaluation in patients with HCC [PR/SD (n = 8) and PD (n = 9)] treated with Atezo+Bev. (**B**,**C**) Clinical course of two representative cases receiving atezolizumab and bevacizumab (Atezo+Bev) for HCC. Case 1 achieved partial response (PR), while Case 2 showed progressive disease (PD) (**C**) at the initial treatment response assessment. The course of imaging studies (arterial phase of dynamic computed tomography), total CTC counts, RNA gene expression in the CTCs (CDKN2B, GRB2, and PIK3R1), tumor size, and tumor marker (AFP, DCP) values are presented. (**B**) The number of CTCs decreased after 4 months, with a 35% reduction in tumor size. After 12 months, expression of the three genes in CTCs and CTC counts increased. Contrast-enhanced CT scan revealed an increase in tumor size, leading to PD assessment. (**C**) While the number of CTCs decreased after 1 and 3 weeks, it increased after 3 months. Tumor size increased by 36% after 3 months, leading to PD assessment. (**A**) Mann–Whitney test. * *p* < 0.05, ** *p* < 0.01, n.s., not significant.

To monitor changes in TGF-β signaling-related genes in CTCs throughout the extended clinical course of Atezo+Bev in patients with HCC, we investigated the gene expression of CDKN2B, GRB2, and PIK3R1 in CTCs. At 3 weeks, the early time point after Atezo+Bev initiation, the expression of CDKN2B and PIK3R1 was remarkably lower compared to baseline in the PR/SD group, while GRB2 and PIK3R1 were elevated in the PD group (Figure 3A). Furthermore, at 3 weeks and response evaluation, CDKN2B, GRB2, and PIK3R1 expressions decreased in the PR/SD group and increased in the PD group, with significant differences observed between the groups. The clinical course is presented for representative cases. The first patient (Case 1, 79 years old, female) had multiple HCCs (BCLC stage B) and achieved a PR response after 4 months of treatment (Figure 3B). Expression levels of CDKN2B, GRB2, and PIK3R1 in CTCs decreased at 1 and 3 weeks after the initiation of Atezo+Bev treatment. Conversely, the second patient (Case 2, 62 years old, female) with multiple HCCs (BCLC stage C) experienced a PD response after 3 months of treatment (Figure 3C). Gene expression of CDKN2B, GRB2, and PIK3R1 in CTCs increased at 1 and 3 weeks following Atezo+Bev initiation. These findings suggest that changes in the expression of genes associated with the TGF-β signaling pathway, specifically CDKN2B, GRB2, and PIK3R1, in CTCs may be biomarkers reflecting treatment response earlier and more accurately than changes in CTC count in patients with HCC treated with Atezo+Bev.

## 4. Discussion

In this study, CTCs were serially collected from the peripheral blood of patients with HCC undergoing Atezo+Bev treatment. Following CTC enrichment, the number of CD45(−)PanCK(+) CTCs measured by fluorescence-activated cell sorting (FACS) showed changes reflecting the effect of Atezo+Bev treatment at the time of response evaluation. Serially collected RNA derived from CTCs was analyzed using NGS for changes in the 373 genes. Additionally, unsupervised hierarchical clustering analysis of gene expression variation in CTCs classified patients into two clusters. Patients clustered with elevated TGF-β signaling molecules in CTCs had a poor response, while the other cluster with elevated apoptosis signaling molecules exhibited a favorable response to Atezo+Bev. Furthermore, changes in the expression of the TGF-β signaling molecules CDKN2B, GRB2, and PIK3R1 in CTCs accurately predicted the therapeutic response earlier than changes in the number of CTCs.

CellSearch, an immunoaffinity-based CTC separation system using the EpCAM antibody, is approved by the U.S. Food and Drug Administration for breast, colon, and prostate cancer [12]. However, EpCAM expression is limited to about 35% of HCC cases [13], and its downregulation during EMT often allows CTCs to evade detection by CellSearch [14,15]. Boral et al. reported that, in patients with breast cancer, the number of CD45(−)PanCK(+)CTCs detected by FACS was approximately three times higher than that collected by CellSearch [16]. Likewise, in a previous study, we isolated CTCs from patients with HCC using a microcavity array system, with the number of detected CTCs being lower than that observed in the current study [8]. RosetteSep, employed in this study, utilizes a biophysical property-based enrichment method that integrates immunoaffinity and density centrifugation to separate CD45(+) leukocytes. In addition, the enriched CTCs obtained through this method are label-free, simplifying downstream separation processes [14]. Given these methodological differences in CTC detection, it is reasonable to expect that the number of CTCs measured by FACS after CTC enrichment in this study would tend to be higher than previously reported HCC cases using CellSearch [17,18].

CTCs are live cells that have intravasated into the bloodstream and are likely derived from invasive and potentially drug-resistant subclones [19]. In patients with breast cancer, Kwan et al. performed digital PCR analysis using RNA from CTCs and found that changes in expression of 6-gene sets after 3 weeks of treatment were associated with rapid disease progression within 120 days [19]. Similarly, in patients with HCC, the number of CTCs tends to correlate with tumor volume, and after surgical resection, a significant decrease in the number of CTCs has been observed on postoperative days 7–10 [14,20,21]. These findings indicate that the early changes in gene expression of CTC-derived RNA after 3 weeks of Atezo+Bev treatment in this study may be attributed to the intravasation of treatment-resistant tumor cell subclones. In addition, it was suggested that the number of CTCs during Atezo+Bev treatment may reflect the tumor volume at the time of collection.

In the tumor microenvironment (TME), TGF-β activation and signaling promote disease progression by stimulating EMT, angiogenesis, and immunosuppression [22,23,24]. High TGF-β expression in TME correlates with poor clinical outcomes and increased metastatic potential across various tumor types [23,25]. TGF-β1 induces the expression of immune checkpoints such as programmed cell death protein 1 (PD-1) and cytotoxic T-lymphocyte associated protein 4 (CTLA4) on T cells, thereby attenuating T cell-mediated anti-tumor immune surveillance [26,27]. Analysis of the Cancer Genome Atlas (TGCA) dataset of HCC samples revealed that clusters with highly activated TGF-β signatures had more pronounced immune exhaustion and poorer prognosis, suggesting that ICI therapy may be less effective [27,28]. Therefore, the enrichment of the TGF-β signaling pathway in CTCs during Atezo+Bev treatment in the non-responder group in this study may indicate that activation of TGF-β signaling in primary HCC contributes to ICI resistance through immune exhaustion and increased CTC numbers by promoting EMT.

In the cancer immune cycle, activated effector T cells induce apoptosis of target cancer cells via perforin and granzyme-mediated or Fas-mediated pathways [29,30]. This process is often impaired due to interactions between PD-1 and PD-L1; however, ICI therapy activates an anti-tumor T-cell response, leading to the apoptosis of cancer cells [30,31]. In patients with breast cancer treated with neoadjuvant systemic therapy, apoptotic disseminated tumor cells (DTCs) in bone marrow were detected more frequently in patients with partial or complete remission compared with those with stable disease [32]. In the present study, enrichment of apoptosis signaling pathway-related genes was observed in CTCs of the responder group, potentially reflecting the anti-tumor effect of Atezo+Bev on primary HCC.

## 5. Conclusions

Our study, utilizing NGS analysis of CTC-derived RNA collected serially from patients with HCC during Atezo+Bev therapy, showed that changes in TGF-β signaling and apoptotic signaling pathways were correlated with treatment response. Specifically, alterations in the expression of genes related to TGF-β signaling, including CDKN2B, GRB2, and PIK3R1, in CTCs emerged as potential novel biomarkers for predicting early therapeutic response to Atezo+Bev in patients with unresectable HCC.

## Figures and Tables

**Table 1:** Antibodies used in this study.

Target	Application	Target Species	Host Species	Clone	Company	Catalogue No.
CD45	FCM	Human	Mouse	2D1	BioLegend	368516
pan-Cytokeratin	FCM	Human	Mouse	C-11	Cayman Chemical	10478

**Table 2:** Primers used in this study.

Gene	Species	Dye	Company	Catalogue No.
CDKN2B	Human	FAM	ThermoFisher Scientific	Hs00793225_m1
GRB2	Human	FAM	ThermoFisher Scientific	Hs00157817_m1
PIK3R1	Human	FAM	ThermoFisher Scientific	Hs00933163_m1
HPRT1	Human	FAM	ThermoFisher Scientific	Hs02800695_m1

**Table 3:** Characteristics of patients with hepatocellular carcinoma and healthy donors.

Characteristics	HCC (*n* = 44)	Healthy Donors (*n* = 10)
Age, median (IQR), years	75 (69–80)	71 (56–77)
Sex, male/female, n	31/13	7/3
Etiology, HBV/HCV/NBNC, n	3/15/26	—
PLT, ×10^9^/L, median (IQR)	151 (119–215)	252 (121–270)
PT, INR, median (IQR)	1.07 (0.99–1.26)	0.98 (0.88–1.00)
ALB, g/dL, median (IQR)	3.5 (3.1–3.8)	4.1 (3.8–4.4)
T-bil, g/dL, median (IQR)	0.8 (0.6–1.2)	0.9 (0.6–1.1)
ALT, IU/L, median (IQR)	25 (15–34)	22 (12–22)
Child-Pugh class, A/B/C, n	33/10/1	
AFP, ng/mL, median (IQR)	12.2 (4.1–117)	—
DCP, mAU/mL, median (IQR)	146 (24–1356)	—
Maximum tumor size, cm, median (IQR)	3.0 (1.6–6.7)	—
Number of tumors, 1/2/3+, n	15/4/25	—
Vascular invasion, absent/present, n	35/9	—
Extrahepatic metastasis, n		
None	36	—
Lymph node	3	—
Bone	1	—
Lung	1	—
Lung, Bone	2	—
Lymph node, Bone, Adrenal gland	1	—
BCLC stage, 0/A/B/C, n	6/6/16/16	—

Abbreviations: AFP, α-fetoprotein; BCLC stage, Barcelona Clinic Liver Cancer stage; DCP, des-gamma-carboxy prothrombin; IQR, interquartile range; NBNC, nonB-nonC.

**Table 4:** List of 373 gene NGS panel associated with stem cell potency, epithelial–mesenchymal transition, HCC progression, and cancer immunotherapy.

Stem Cell Potency								
BMI1	COL2A1	FGF5	GRIN1	ISL1	MYF5	PDX1	SEMA3A	TERT
CALB1	COMMD3	FLT1	GSX2	JARID2	MYOD1	PECAM1	SERPINA1	TFCP2L1
CD34	CRABP2	FOXA2	HAND1	KIT	NANOG	PODXL	SERPINB3	THY1
CD9	CRK	FOXC1	HBB	KLF4	NEUROD1	POU5F1	SET	TRIM24
CDH5	CTLA4	FOXD3	HBZ	LAMB1	NEUROG1	PROM1	SFRP2	WT1
CDK2	CTNNB1	GABRB3	HESX1	LAMC1	NODAL	PTEN	SKIL	XIST
CDK4	DDX4	GAL	HGF	LEFTY1	NOG	PTF1A	SMARCAD1	ZFHX3
CDKN1A	DES	GATA4	HNF4A	LEFTY2	NPPA	RAF1	SOX17	ZFP42
CDKN2A	DNMT3B	GATA6	HOXB1	LHX5	NR5A2	REST	STAT1	ZIC3
CDKN2B	E2F1	GBX2	IAPP	LIFR	NR6A1	RFX4	STAT3	
CDX2	ENO2	GCM1	IFITM1	LIN28A	OLIG2	RIF1	SYCP3	
CDYL	EOMES	GDF3	IFITM2	MEIS1	ONECUT1	RNF112	SYP	
CGB	EPCAM	GFAP	IGF2BP2	MNX1	OTX1	RUNX2	TAT	
COL1A1	ERBB2	GJA9	IL6ST	MUC1	PAX4	SALL1	TCF7L1	
COL1A2	ESX1	GRB7	ILK	MYC	PAX6	SALL4	TDGF1	
Epithelial–mesenchymal transition								
AHNAK	COL3A1	FGFR3	IL1RN	KRT18	MMP3	RELA	SRC	TSPAN13
AKT1	COL5A2	FN1	INS	KRT19	MMP9	RGS2	STEAP1	TWIST1
AKT2	CYCS	FOXC2	IPO8	KRT4	MSN	RHOA	TCF3	VCAN
ANGPT2	DESI1	FYN	ITGA5	KRT5	MST1R	SERPINE1	TCF4	VIM
AXL	DSC2	FZD7	ITGAV	KRT7	NES	SMAD2	TFPI2	VPS13A
BMP1	DSP	GEMIN2	ITGB1	KRT72	NOTCH1	SMAD4	TGFA	WNT11
BMP7	EGFR	GNG11	JAG1	KRT8	NUDT13	SMAD7	TGFB1	WNT5A
BRAF	ELK1	GRB2	JUN	LAMA1	OCLN	SNAI1	TGFB2	WNT5B
CALD1	ERBB3	GSC	KRAS	LEF1	PDGFRB	SNAI2	TGFB3	ZEB1
CAMK2N1	ESR1	GSK3B	KRT1	MAP1B	PLEK2	SNAI3	TGFBR1	ZEB2
CAV2	F11R	HMBS	KRT1	MAX	PTK2	SOX10	TGFBR2	
CDC42	FADD	IGF1	KRT10	MDM2	PTP4A1	SOX2	TIMP1	
CDH1	FGFR1	IGF1R	KRT13	MITF	RAC1	SPARC	TMEFF1	
CDH2	FGFR2	IGFBP4	KRT14	MMP2	RB1	SPP1	TMEM132A	
HCC progression								
ABCC5	BRIX1	CYSLTR1	FGFBP1	KDR	MRPL19	POLR2A	RET	VEGFA
ABL1	CASC3	CYSLTR2	FGFR4	LRRC23	MUC16	POP4	RPL37A	WNT1
ACTC1	CASP8	DVL1	FOS	LTB4R	NFKB1	PSAT1	RUNX3	XIAP
AFP	CASP9	EEF1A1	FZD1	LTB4R2	NFKB2	PSMC4	SHC1	YAP1
ALB	CCND1	EEF1A2	GADD45A	MAP2K1	NFKBIA	PTGS1	SOCS3	
ALOX5	CCND2	EIF2B1	GPC3	MAP3K5	NRAS	PTGS2	SOS1	
APC	CCND3	ELF1	HRAS	MAPK1	OAS2	PTK2B	T	
ASGR1	CDKN1B	FGF19	IFI44L	MAPK14	PDCD1	PUM1	TBXT	
BAX	CEACAM5	FGF2	ITGA2B	MAPK3	PES1	PYCARD	TNFRSF10B	
BCAR1	CEACAM8	FGF23	ITGB3	MAPK8	PIK3CA	RASSF1	TNFSF10	
BID	CELA1	FGF4	KAT6A	MET	PIK3R1	RELN	TP53	
Cancer immunotherapy								
APOBEC3G	CCL2	CCNE1	CD8A	CXCL9	IRF1	IRF5	PTPRC	
BCL2	CCL3	CD274	CHUK	FAS	IRF2	IRF6	TLR4	
BCL2L1	CCL4	CD28	CX3CL1	FASLG	IRF3	IRF7		
BCL2L11	CCL5	CD4	CXCL1	IKBKB	IRF4	IRF9		
APOBEC3G	CCL2	CCNE1	CD8A	CXCL9	IRF1	IRF5	PTPRC	

**Table 5:** Characteristics of patients with hepatocellular carcinoma treated with Atezolizumab + Bevacizumab.

Characteristics	Atezolizumab + Bevacizumab (n = 22)
Age, median (IQR), years	69 (62–79)
Sex, male/female, n	17/5
Etiology, HBV/HCV/NBNC, n	3/8/11
PLT, ×10^9^/L, median (IQR)	154 (110–204)
PT, INR, median (IQR)	1.13 (0.98–1.24)
ALB, g/dL, median (IQR)	3.5 (3.0–3.9)
T-bil, g/dL, median (IQR)	1.0 (0.6–1.4)
ALT, IU/L, median (IQR)	26 (16–38)
Child–Pugh class, A/B, n	17/5
AFP, ng/mL, median (IQR)	16.5 (4.4–404)
DCP, mAU/mL, median (IQR)	261 (24–1452)
Maximum tumor size, cm, median (IQR)	3.8 (2.0–7.2)
Number of tumors, 1/2/3+, n	4/1/17
Vascular invasion, absent/present, n	15/7
Extrahepatic metastasis, n	
None	16
Lymph node	2
Bone	1
Lung	1
Lymph node, Lung	1
Lymph node, Bone, Adrenal gland	1
BCLC stage, A/B/C, n	1/10/11
Prior systemic therapy, n	
None	13
Sorafenib	1
Lenvatinib	6
HAIC	1
Lenvatinib, HAIC	1
Observation period, median, days	305

Abbreviations: AFP, α-fetoprotein; BCLC stage, Barcelona Clinic Liver Cancer stage; DCP, des-gamma-carboxy. Pro-thrombin; HAIC, hepatic arterial infusion chemotherapy; IQR, interquartile range; NBNC, nonB-nonC.

## Data Availability

The data of the current study are available from the corresponding author upon reasonable request.

## Abbreviations

AEs: adverse events; Atezo+Bev, atezolizumab plus bevacizumab; CTCs, circulating tumor cells; EMT, epithelial–mesenchymal transition; HBsAg, surface antigen of hepatitis B virus; HBV, hepatitis B virus; HCC, hepatocellular carcinoma; HCV Ab, anti-hepatitis C virus antibody; ICI, immune checkpoint inhibitor; NBNC, non-B-non-C tumor; OS, overall survival; PBS, phosphate-buffered saline; PD, progressive disease; PFS, progression-free survival; PR, partial response; SD, stable disease.
